# Brown Tumor: An Uncommon Manifestation of Severe Hyperparathyroidism

**DOI:** 10.7759/cureus.89067

**Published:** 2025-07-30

**Authors:** Muhammad Imran, Samuel Spitalewitz, Bakhtyar Khan, Anthony Odubanjo

**Affiliations:** 1 Nephrology, Brookdale University Hospital Medical Center, Brooklyn, USA

**Keywords:** brown tumor, end-stage renal disease (esrd), parathyroidectomy, patient non-adherence, severe hyperparathyroidism

## Abstract

Brown tumor is now an uncommon form of osteitis fibrosa cystica. It results from an imbalance in bone remodeling, which leads to osteolytic lesions. This process, marked by microfractures and discoloration, is triggered by persistently elevated parathyroid hormone levels in end-stage renal disease (ESRD) patients with tertiary hyperparathyroidism. Our case highlights a large brown tumor in the maxilla causing significant facial deformity.

A 34-year-old female patient with ESRD had multiple admissions to our facility for various reasons. She was non-adherent to her dialysis treatment regimen and medications. She had a facial mass that had doubled in size over a year. Laboratory results showed elevated calcium and phosphorus levels and extremely elevated parathyroid hormone (PTH) levels. A computed tomography (CT) scan showed an exophytic mass arising from the anterior left maxilla, suggestive of a brown tumor. A sestamibi parathyroid scan and neck sonogram did not confirm hyperplastic parathyroid glands. Nevertheless, a biopsy of the mass showed multinucleated giant cells and stromal spindle cells consistent with a brown tumor. The patient would have benefited from a parathyroidectomy, but she refused and signed out against medical advice.

Brown tumors are now uncommon in the USA due to effective medical management of bone mineral disorders associated with ESRD and efficient renal replacement therapy. However, our case emphasizes that brown tumors may occur in ESRD patients who do not adhere to their treatment regimen.

## Introduction

The pathophysiology of secondary hyperparathyroidism associated with chronic renal insufficiency is well described, and the treatment is well established. Treatment options are medical management and surgery for resistant cases to control severe hyperparathyroidism and its associated consequences. The number of patients requiring surgical intervention has decreased due to the efficacy of phosphorus binders, vitamin D analogs, and calcimimetics. However, as with all interventions, patient’s adherence is mandatory, without which severe secondary hyperparathyroidism can be progressive and disabling with severe osteitis fibrosa cystica, fractures, disfigurement, and a multitude of other untoward effects including severe vascular calcifications, anemia, calciphylaxis, immune dysfunction, heart failure, and increased risk of cardiovascular morbidity and mortality [1-3].

Brown tumor is a type of osteitis fibrosa cystica, a form of bone mineral disease associated with uncontrolled hyperparathyroidism, which results from an imbalance in bone remodeling. In the later phases of the remodeling process, there is an increase in osteoclast activity not balanced by an increase in osteoblast activity, resulting in osteolytic lesions. The remodeling process may cause hemorrhage, which results in the release of hemosiderin, causing a discoloration that explains the name “brown tumor.” These lesions occur most commonly in the maxilla and mandible but may involve any bone. They may be invasive but are not malignant [4-8].

We present a case of a dialysis-dependent patient with brown tumors due to uncontrolled hyperparathyroidism who was resistant to our efforts to control her severe hyperparathyroidism.

## Case presentation

We present a 34-year-old female patient with a past medical history of hypertension, seizure disorder, heart failure, and end-stage renal disease (ESRD) of unknown etiology, who had been on maintenance hemodialysis for three years and was non-adherent to treatment sessions and medications, which resulted in multiple presentations to the emergency department for various reasons. She frequently signed out against medical advice. Other pertinent medical history includes tertiary hyperparathyroidism, systolic heart failure with an ejection fraction of 30%, recurrent pericardial effusions, pulmonary hypertension, and a seizure disorder due to traumatic brain injury.

**Figure 1:**
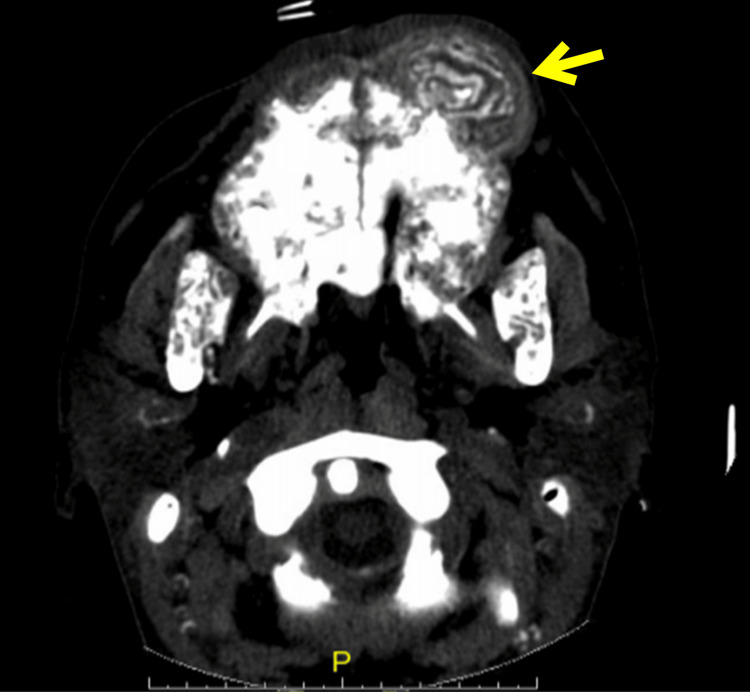
CT scan of the face and soft tissues showing a left maxillary mass (arrow) CT: computed tomography

Her most recent admission was in July 2019 for respiratory distress secondary to pulmonary edema due to fluid overload after missing hemodialysis. Her examination was notable for a heart rate of 115 beats/minute, blood pressure of 150/98 mmHg, visible jugular venous distension, an S3 gallop, bilateral rales, bilateral dependent edema, and facial swelling and deformity. Detailed examination of the facial swelling was notable for a left homogenous maxillary mass, hard, nodular, non-tender, non-pulsatile, around 3.5 × 3.0 cm, noted about more than a year ago, gradually increasing in size, with no discharge noted over the mass. Laboratory data were pertinent for a hemoglobin of 8.3 g/dL, calcium of 10.8 mg/dL, phosphorus of 6.5 mg/dL, and an intact parathyroid hormone (iPTH) level of 3,645 pg/mL (normal range: 14-64 pg/mL) (Table 1), compatible with tertiary hyperparathyroidism. A two-dimensional echocardiogram again showed a moderate pericardial effusion and an ejection fraction of 30%. The patient’s symptoms of volume overload improved after emergency hemodialysis. Although previously prescribed, she received cinacalcet and sevelamer for the management of her severe bone mineral disorder. A maxillofacial computed tomography (CT) scan showed widespread heterogeneity of the osseous structures, probably related to renal osteodystrophy with an exophytic mass arising from the anterior left maxilla, measuring approximately 4.3 × 3.6 × 2.7 cm, most likely representing a brown tumor (Figure 1). This was subsequently confirmed by a left maxillary bone biopsy, which showed multinucleated giant cells and stromal spindle cells, consistent with “brown tumor.” In preparation for a possible parathyroidectomy, imaging studies were done, which included a sestamibi parathyroid scan, which did not show hyperplastic parathyroid glands. A neck sonogram was also unrevealing. She developed the described severe hyperparathyroidism due to non-adherence to the medical regimen and frequently missed dialysis treatments. It was determined by psychiatric consultation that the patient was competent to make medical decisions. After a thorough explanation that her ongoing severe hyperparathyroidism and its multiple complications would improve with parathyroidectomy, she refused and left against medical advice.

**Table 1:** Parathyroid hormone, calcium, and phosphorus levels over different visits iPTH: intact parathyroid hormone

Test	July 2018	October 2018	February 2019	July 2019
Calcium (mg/dL)	8.3	9.2	8.6	10.8
Phosphorus (mg/dL)	6.8	5.9	6.3	6.5
iPTH (pg/mL)	2,714	2,212	4,668	3,645

## Discussion

Non-adherence to hemodialysis and the medications prescribed leads to multiple complications, including osteitis fibrosa cystica, a form of renal osteodystrophy. Additionally, a brown tumor, a form of osteitis fibrosa cystica, has been widely reported in patients with high bone turnover due to uncontrolled hyperparathyroidism and is characterized by deformity and bone pain [9,10].

Our patient had severely elevated parathyroid hormone (PTH) levels and elevated calcium and phosphorus levels. This is the result of persistent hyperparathyroidism, which in our patient was caused by non-compliance with treatment [11]. The diagnosis of tertiary hyperparathyroidism in our patient was thus established based on clinical, laboratory (increased PTH, calcium, and phosphorus levels), and pathology findings. However, imaging did not confirm the diagnosis, since the sensitivity of sestamibi scan and neck ultrasound for detecting hyperplastic parathyroid tissue is 50% and 57%, respectively [12,13].

The pathogenesis of hyperparathyroidism in renal failure is multifactorial and is associated with elevated fibroblast growth factor 23 (FGF23), reduced 1α,25(OH)2D levels, hyperphosphatemia, hypocalcemia, and reduction of vitamin D receptors (VDR) and calcium-sensing receptors (CaSR) in the parathyroid gland [14,15]. These all cause an enhanced rate of synthesis and release of PTH [14]. This results in excessive osteoclastic activity that leads to demineralization of bone matrix, thus releasing calcium. Fibroblasts and groups of hemosiderin-loaded macrophages proliferate to fill in the osteolytic gaps in the matrix of the bone, thus replacing the gaps with loose connective tissue and hemosiderin deposits, giving brown tumors their characteristic color [8-10,16].

Tertiary hyperparathyroidism, characterized by hypercalcemia and hyperphosphatemia, is the most advanced form and is caused by autonomous functioning of the parathyroid gland [6].

The medical treatment of tertiary hyperparathyroidism and brown tumor is the correction of the underlying hyperparathyroidism with phosphate binders and calcium-sensing receptor antagonists. If the patient is unresponsive to medical therapy, a parathyroidectomy is warranted. Ideally, our patient would have benefited from parathyroidectomy. However, in unreliable patients who are non-compliant with treatment, as seen in our case, surgery may not be advisable since there is a need for intense postoperative care to control the frequent severe postoperative hungry bone syndrome with life-threatening hypocalcemia. In either case, she refused surgery. Excision of the brown tumor via curettage or local radiotherapy is recommended if it is a large, destructive tumor or if it significantly compromises the quality of life of the patient [8,17-20]. The patient refused this intervention as well and almost predictably signed out against medical advice.

## Conclusions

Effective treatment to prevent secondary hyperparathyroidism is widely available, and therefore, tertiary hyperparathyroidism and associated brown tumors are now uncommon in the USA. However, these tumors may still occur in end-stage renal disease (ESRD) patients who are non-adherent to therapy. Our case contributes to the literature by highlighting the need for ongoing surveillance of ESRD patients and ensuring their adherence to the medical regimen.
